# The Relationships Among Periodontitis, Pneumonia and COVID-19

**DOI:** 10.3389/froh.2021.801815

**Published:** 2022-01-21

**Authors:** Mikaela Brock, Shaima Bahammam, Corneliu Sima

**Affiliations:** ^1^Department of Oral Medicine, Infection, and Immunity, Harvard School of Dental Medicine, Boston, MA, United States; ^2^Department of Dentistry, King Fasial Specialist Hospital and Research Center, Riyadh, Saudi Arabia

**Keywords:** periodontitis, pneumonia, COVID-19, SARS-CoV-2, oral-systemic, respiratory distress syndrome

## Abstract

Periodontitis is a chronic inflammatory disease of the supporting structures of the teeth that affects approximately half of adults 30 years and older. There is increasing interest in the direct and indirect relationships between periodontitis and systemic diseases, including respiratory diseases. The aim of this study was to assess the evidence on links among periodontitis, pneumonia, and COVID-19. Oral and periodontal bacteria may be linked to respiratory disease directly by aspiration of pathogens into the lungs causing pneumonia. As SARS-CoV-2 began to spread worldwide in 2020, questions have arisen of how periodontal disease may also be connected to SARS-CoV-2 infection and severity, including potential replication and dissemination of the virus from periodontal pockets. Some proposed mechanisms include the oral cavity acting as a reservoir or point of entry for SARS-CoV-2, overgrowth of periodontal pathogens, and increased production of proinflammatory cytokines. Due to potential links between periodontal disease and respiratory infections like pneumonia and SARS-CoV-2, oral hygiene and management of periodontitis remain essential to help reduce infection and transmission of SARS-CoV-2.

## Introduction

Periodontitis is a non-reversible chronic inflammatory disease of the supporting structures of the teeth, including the alveolar bone, cementum, gingiva, and periodontal ligament. Periodontitis pathogenesis is marked by altered host-biofilm interactions that lead to overgrowth of periodontal pathogens, particularly *Porphyromonas gingivalis, Treponema denticola*, and *Tannerella forsythia*, with subsequent loss of periodontal tissues and infection driven by anaerobes [1]. The relative contribution of the innate and acquired immunity's inefficient capacity to resolve inflammation to the onset and progression of periodontitis is not fully understood [2]. Individual predisposition plays a role in the development of periodontitis, and therefore the importance of personalized early intervention for improved periodontal and systemic health cannot be understated.

Approximately half of adults in the United States aged 30 years and older have periodontitis, and the disease prevalence increases with age. 34% of adults have moderate periodontitis, and 8% of adults have severe periodontitis [3]. Globally, approximately 10% of the population is burdened by severe periodontitis, and periodontal diseases overall have a prevalence of approximately 20–50% [4, 5]. This heavy global burden prompted an increasing interest in the relationship between periodontitis and systemic diseases, such as cardiovascular disease, diabetes, obesity, and respiratory infections. Periodontitis is associated with systemic diseases in both direct and indirect ways. Periodontal pathogens can directly distribute throughout the body, or they can indirectly affect systems distant from the oral cavity by stimulating local host immune responses associated with leakage of inflammatory mediators in the circulation and systemic pro-inflammatory priming of the immune system [6]. The relationship between poor control of oral biofilms and systemic diseases, particularly respiratory infectious diseases, suggests the possibility of a bidirectional link between periodontitis and pneumonia.

In December 2019, an unknown pneumonia began spreading around Wuhan, China, which was later identified as Coronavirus disease 2019 (COVID-19) caused by severe acute respiratory syndrome coronavirus 2 (SARS-CoV-2). This virus quickly spread worldwide, with symptoms such as cough, dyspnea, fever, and presentations varying between asymptomatic and acute respiratory distress syndrome (ARDS) and death. The World Health Organization (WHO) announced SARS-CoV-2 as a Public Health Emergency of International concern on Jan 30, 2020, later announced it as a pandemic in March 2020 [7]. The spread of SARS-CoV-2 via droplet transmission raised questions about the relationship between the virus and the oral cavity. Due to previous evidence of relationships between oral biofilms and pneumonia, in particular aspiration and ventilator-associated pneumonias, there is increasing interest in potential bidirectional links between periodontitis and COVID-19.

**Figure 1:**
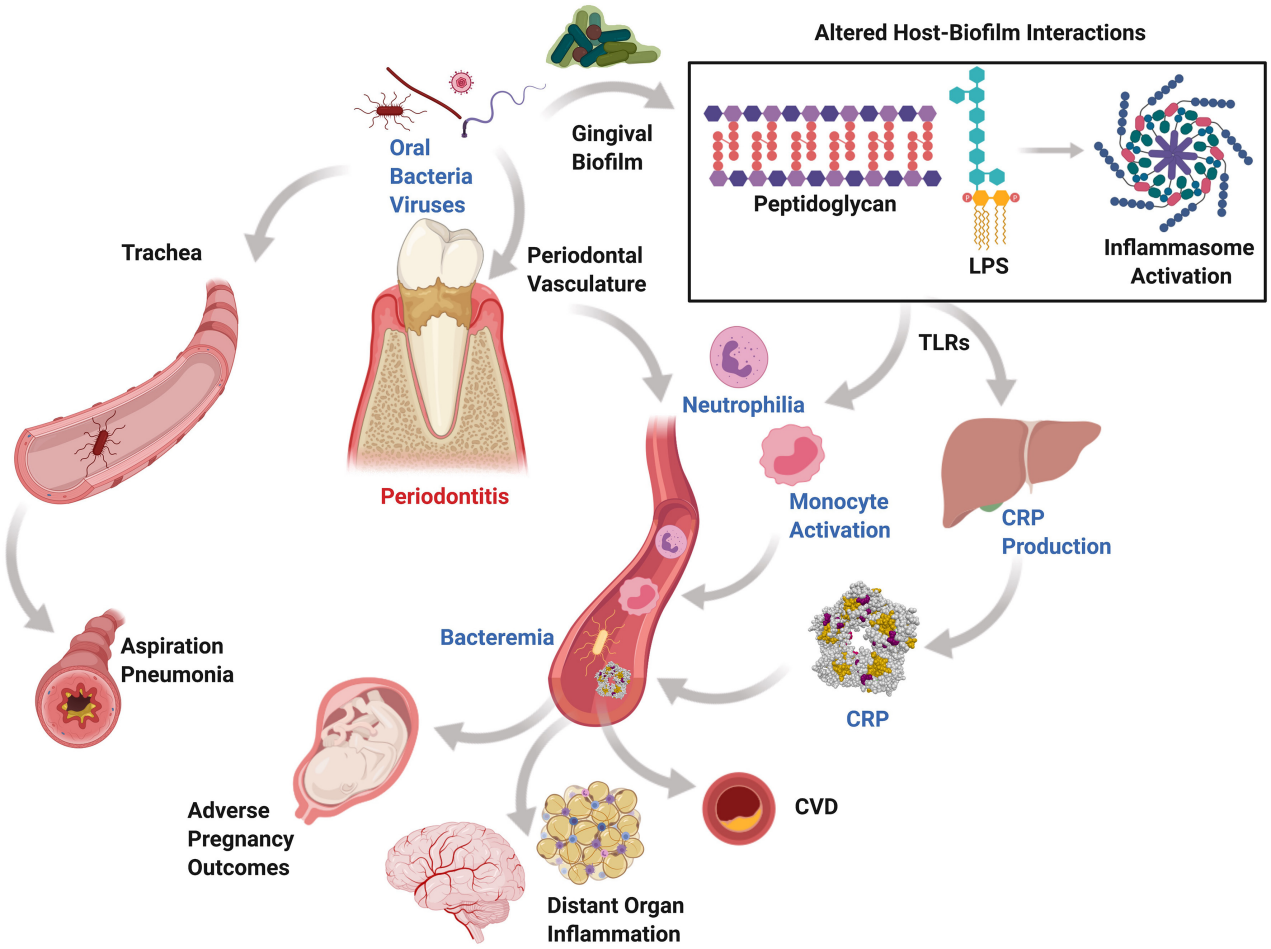
Mechanisms of periodontal systemic connections. Periodontitis, a chronic inflammatory condition affecting tooth-supporting structures and resulting from altered host-biofilm interactions, generates periodontal pockets that harbor oral anaerobic bacteria and viruses. These can be released systemically through the highly vascularized pocket granulation tissue or through aspiration via the oropharynx in those at risk and during therapeutic interventions. Circulatory release of proinflammatory cytokines, bacteria, and their byproducts may be associated with bacteremia, circulating leukocyte priming, low-grade inflammation, and ectopic deposition in distant organs. Similarly, aspirated anaerobic bacteria can colonize the lungs and lead to aspiration pneumonia.

Periodontitis influences the initiation and progression of multiple non-oral systemic diseases. Generally, periodontal disease is underestimated by patients and medical providers; however, the surface area of ulcerated periodontal pockets in a periodontitis patient were estimated to be up to the size of the palm of a hand, which is considered a sizeable inflammatory area that would mandate an urgent medical intervention if it was in other sites of the body [8]. There are two main mechanisms in which periodontal pathogens, or other pathogens harbored in periodontal pockets, could contribute to systemic diseases via direct and indirect routes (Figure 1). The direct route is paved by pathogens that enter the systemic circulation through the ulcerated periodontal pocket lining or during surgical procedures, invasive dental procedures, or daily activities disrupting the periodontal pocket leading to bacteremia [9], which leads to migration of microbes to distant target organs [10, 11]. The indirect route may be driven the bacterial byproducts and the host inflammatory immune response to periodontal pathogens that may play a significant role in the pathogenesis of systemic inflammatory diseases [12, 13]. It is likely that both mechanisms simultaneously impact the pathogenesis of systemic diseases. For example, in atherogenesis, *Porphyromonas gingivalis*, a significant pathogen in periodontal disease, has demonstrated the ability to interact with the endothelial surface and cause damage to the integrity of the endothelium. At the same time, the elevated level of inflammatory cytokines and serum C-reactive protein (CRP) in periodontitis patients plays a role in endothelial dysfunction [14]. It has been found that a statistically significant excess risk exists for atherosclerotic cardiovascular disease in patients with periodontitis, independent of other co-morbidities [15]. The released periodontitis-associated bacterial antigens such as lipopolysaccharides (LPS) and host-derived matrix metalloproteinases (MMPs) stimulate an immune response leading to production of proinflammatory cytokines such as interleukins (IL-1 beta, IL-2, IL-6, and IL-8) and CRP that mediate multiple systemic events.

Periodontitis has also been linked to pregnancy adverse effects in which periodontal bacteria and their endotoxins may spread to the uterine cavity from the blood leading to intrauterine infection or inflammation-mediated contractions potentially leading to preterm birth [16]. This subclinical infection of the uterus results in PGE-2 production responsible for preterm birth through stimulating muscle contraction and severe neonatal sequelae, including periventricular leukomalacia, cerebral palsy, and many other adverse effects. Similarly, the host immune response associated with periodontitis has been proven to affect the glycemic control of diabetic patients. Importantly, several lines of evidence found that the treatment of periodontitis leads to improved glycemic control in both prediabetic and diabetic patients with long-term HbA1c reduction of 0.3–0.5% [17–20]. Periodontitis treatment has also been found to improve surrogate markers for cardiovascular disease, where periodontal treatment resulted in short term systemic inflammation and endothelial dysfunction, but endothelial function improved after 6 months of therapy [13]. A separate study looking at the incidence of stroke in periodontitis patients found that periodontal disease was independently associated with cardioembolic and thrombotic strokes, and regular dental care was associated with lower stroke risk [21]. These results emphasize the connection between periodontal disease and overall health, which provides the basis for the importance of the management of periodontal diseases.

With increasing interest in the relationships between periodontitis and systemic diseases, this brief narrative review aims to discuss the links between periodontitis and respiratory infections such as pneumonia, as well as visit potential links to COVID-19.

## Periodontitis and Pneumonia

Lower airway infections such as pneumonia are one group of conditions that have increasing evidence of direct and indirect links to periodontitis. Many oral pathogens have been implicated in lung infections, with some overlap with periodontal disease, such as *Porphyromonas gingivalis*. One study showed that respiratory pathogens isolated from the same patients in both dental plaque and bronchoalveolar fluid were genetically identical [22]. Pneumonia is a microbial infection that results in inflammation of the lungs. Bacteria that cause pneumonia can enter the lungs by aspiration from the oral and nasal cavities, and this is often seen in patients with decreased levels of consciousness or that have difficulty swallowing.

Aspiration pneumonia can occur due to infection with commensal oral bacteria, whereas nosocomial infection occurs due to the introduction of foreign bacteria into the oral cavity that can be integrated into plaque [23]. A review by Sabharwal et al. showed that while oral bacteria can cause pneumonia, the evidence is inconsistent, and larger-scale studies are needed to further elucidate the relationship between oral pathogens and pneumonia. While multiple studies have been conducted to compare oral hygiene protocols for reducing the risk of aspiration pneumonia in different settings, it is important to note that many patients are admitted to hospitals and nursing homes with increased plaque buildup due to inadequate oral care [23]. Although these studies have not documented the diagnoses of periodontal diseases, it is reasonable to assume that the incidence of periodontal inflammation is high in these cohorts.

A Cochrane review looked into oral healthcare measures and the prevention of nursing home acquired pneumonia. Nursing home-acquired pneumonia occurs due to aspiration of oral bacteria into the lungs, as well as the inability of a patient to resolve the infection. This review found low-quality evidence that professional oral care could potentially reduce the risk of mortality associated with nursing home acquired pneumonia compared to standard oral care [24]. However, due to study design problems and a small number of studies, this evidence should only be considered with caution. Future research is recommended to further investigate this connection. The proposed mechanism of this professional oral care was mechanical removal of plaque and the use of oral antiseptics to reduce the buildup of plaque and thus microbial deposits in elderly patients. Gram-negative bacilli bacteria were detected at higher rates in frail elderly, which further emphasized the importance of oral hygiene [25, 26]. The use of a chlorhexidine (CHX) rinse daily or weekly improved oral conditions, and manual brushing also reduced colonization of oral microbes in residents of nursing homes and other long term care facilities, which could decrease the risk of aspiration of pneumonia-causing bacteria [24].

Similarly, critically ill patients receiving mechanical ventilation for at least 48 hours are at risk of developing ventilator-associated pneumonia (VAP). A Cochrane review that assessed the effect of oral hygiene on the incidence of VAP, found high level of evidence from 18 randomized controlled trials (2451 participants, 86% adults) that oral health care involving the use of CHX mouth rinse or gel reduced the risk of VAP from 24 to 18%, with 1 out of every 17 patients receiving this beneficial outcome [27]. However, no significant differences in outcomes for mortality, duration of ventilation, or duration of intensive care unit stay were found. It is thought that infection is more likely to occur in these patients due to how rapidly plaque can build up in critically ill patients due to inadequate oral hygiene. The actual mechanism of infection is thought to be either due to the endotracheal tube serving as a pathway for infection from the oral cavity to the lungs or from microaspiration can occur around the seal of the endotracheal tube.

Periodontitis and respiratory infections share common inflammatory pathogenesis and risk factors, one of which is smoking. Smoking increases the host's susceptibility and risk of infection by inducing immune dysfunction and poor vascularization [28]. Robust evidence exists that smoking is closely associated with acute and chronic respiratory infections such as pneumonia [29]. Smoking is also a significant risk factor and modifier of periodontitis expression. Available evidence suggests that smoking results in alteration in subgingival microbial community and facilitates colonization of periodontal pathogens [30]. Poor oral health in concert with other factors such as smoking could promote the progression and exacerbation of pulmonary disease for those at risk.

Altogether, existing evidence supports the idea that inadequate oral hygiene and increased oral microbial loads may place patients at higher risk of pneumonia, and interventions should be put into place to control the accumulation of oral biofilm. CHX rinses could be considered to control dental biofilms, and aerosols should be minimized in dental procedures to decrease the risk of aspiration. Further research to investigate the link between the severity of periodontal diseases and the risk of respiratory infections.

## Periodontitis and COVID-19

As the COVID-19 pandemic continues, more data builds on the potential relationship between periodontitis and COVID-19. However, this data is not definitive. It is reasonable to hypothesize that similar to aspiration pneumonia, respiratory dissemination of the virus from the oral cavity can promote SARS-CoV-2 infection. Two of the earliest and commonly reported symptoms in SARS-CoV-2 infection are the loss of taste and smell. Early reports on loss of taste and smell during SARS-CoV-2 infection were further supported by findings that oral epithelial cells can replicate the virus [31]. Taste receptor cells (TRCs) on the tongue express ACE2, which is the receptor that SARS-CoV-2 binds to, as well cathepsin, a protease that aids in binding. TRC I, the supporting cells of other TRCs, also express ACE2 and can thus be targeted by SARS-CoV-2. Without these supporting cells, cell death can occur in other TRCs, resulting in decreased taste signals to nerves. This viral uptake of SARS-CoV-2 in TRCs is only one possible route of entry of SARS-CoV-2 in the oral cavity.

In order for the virus to infect a host, it must first enter cells via the ACE2 receptor. Prior to entry, furin and TMPRSS2 cleave the S protein on SARS-CoV-2 into S1 and S2, and S1 binds to ACE2 to enter the cell. ACE2 is expressed in multiple sites in the body, including the lungs, kidneys, heart, liver, and intestines. A study using a COVID-19 mouse model showed that increased expression of ACE2 increased the severity of SARS-CoV-2 infection, indicating that viral entry into the cell is a rate limiting step for virulence [32]. A more recent investigation used two single-cell RNA sequencing datasets of human minor salivary glands and gingiva, and it found that ACE2 and TMPRSS are abundant in the minor salivary glands and oral mucosa, confirming that these tissues can be entry points for SARS-CoV-2 [33].

**Figure 2:**
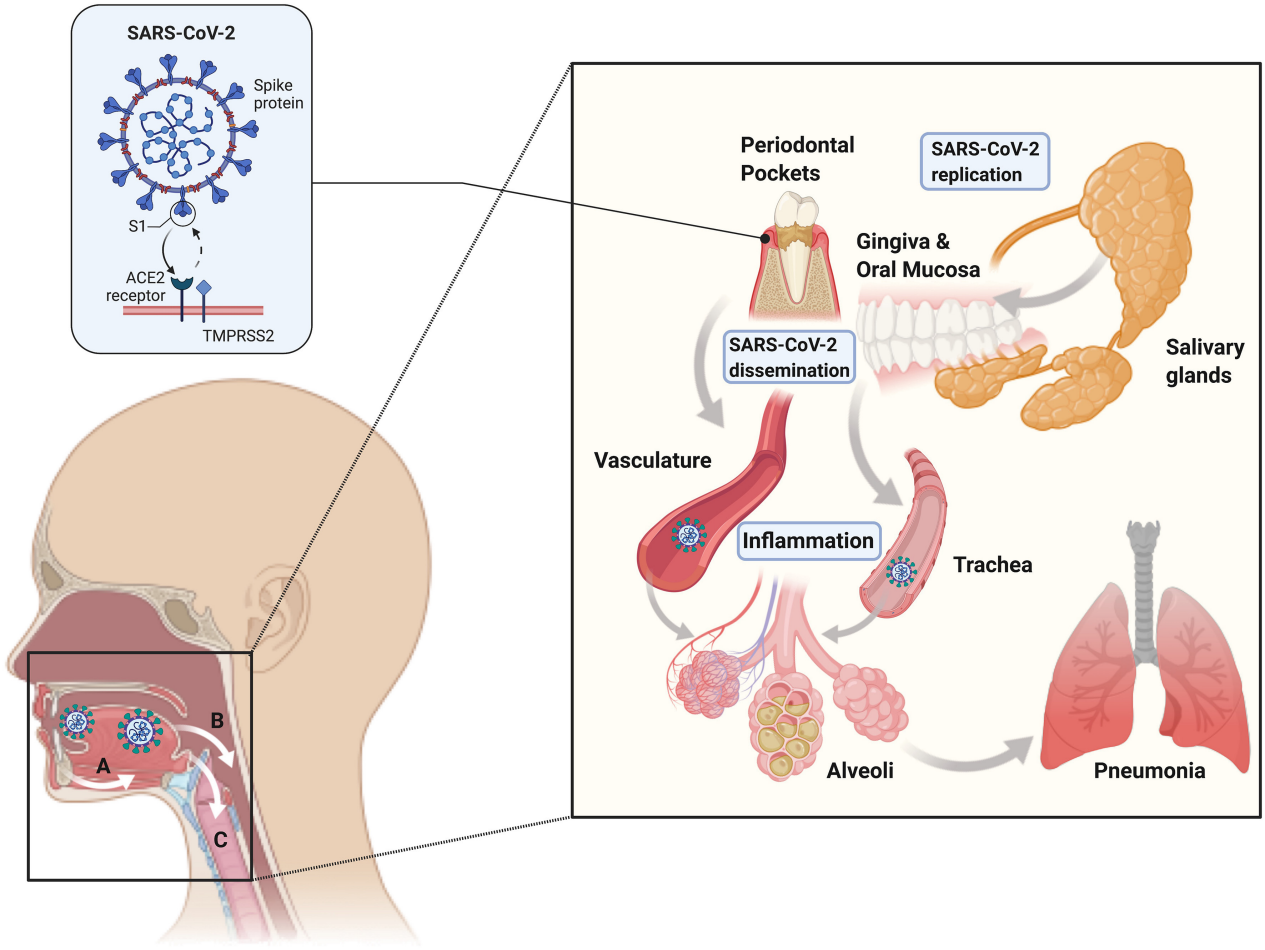
Mechanisms of periodontal-COVID-19 connections. The existing evidence suggests that SARS-CoV-2 can independently replicate in the oral cavity in epithelial cells (including periodontal pockets) and salivary glands (*right inset*). Gingival and mucosal epithelial uptake of SARS-CoV-2 is thought to occur through ACE2-TMPRSS2 receptors (*left inset*). Viral shedding from epithelial surfaces and saliva can lead to systemic dissemination via hematologic **(A)**, digestive **(B)**, and respiratory **(C)** routes. Therefore, the oral cavity can represent a SARS-CoV-2 replication and dissemination reservoir, leading to acute respiratory and lower digestive tract infection.

Additionally, droplet PCR showed SARS-CoV-2 can be detected in the salivary glands, mainly the minor salivary glands. Ducts and acini infected with SARS-CoV-2 also contained replicating viruses, which showed that the minor salivary glands could be a source of SARS-CoV-2 in saliva, where the virus can replicate, survive, and then spread to other individuals and throughout the body. They also showed that the saliva of asymptomatic individuals contained SARS-CoV-2 that could be spread via droplets, and the amount of viral burden in saliva correlated with the presence of viral symptoms [33]. This presence of replicating SARS-CoV-2 in oral tissues supports the idea that the oral cavity may serve as a reservoir for the virus, with the virus's potential to bind to ACE2 receptors in the oral cavity for entry into the host (Figure 2). Thus, oral hygiene and periodontal therapy should be considered to decrease the microbial load in the oral cavity and potentially decrease viral loads to the limit disease spread. Other hypotheses on how periodontitis may be linked to SARS-CoV-2 infection relate to the evidence on periodontitis connections to other respiratory syndromes. One thought is that aspiration of pathogenic periodontal bacteria can increase the expression of ACE2 and lead to the production of inflammatory cytokines. Additionally, it is possible that periodontal bacteria promote the release of inflammatory cytokines that may contribute to the development of cytokine storm and acute respiratory distress syndrome [34].

Recent clinical studies were carried to elucidate the relationship between periodontitis and SARS-CoV-2. In a study conducted at the Postgraduate Institute of Medical Education and Research in Chandigrah, India, 82 SARS-CoV-2 patients underwent periodontal examinations and had blood parameters measured [35]. The study assessed SARS-CoV-2 related outcomes such as SARS-CoV-2 related pneumonia, death, hospital admission, and need for assisted ventilation. They found that these SARS-CoV-2 related outcomes, such as hospital admission, assisted ventilation, and pneumonia increased as the periodontal disease stage increased. Assisted ventilation was highest in patients with Stage III and IV periodontitis. The more severe periodontal disease, the greater the odds of assisted ventilation use, hospital admission, mortality, and acquired SARS-CoV-2 related pneumonia. Approximately 10% of the patients died from SARS-CoV-2, and these patients also had more severe periodontitis. A case control study performed in the state of Qatar evaluated the national electronic health records of 568 patient who had COVID-19 to investigated the association between periodontitis and COVID-19 complications [36]. Out of the 40 patients who suffered from COVID-19 complications, 33 had moderate to severe periodontitis. The calculated odds ratios of having COVID-19 complications in periodontitis patients after adjusting for possible confounders including age, sex, smoking, BMI, diabetes and comorbidities was 6.34 for any complications, 17.5 for death, 5.57 for ICU admission and 7.31 for need for assisted ventilation. A separate case-control study was conducted at the Department of Dentistry at ESIC Medical College in Hyderabad, India that also included periodontal examinations for SARS-CoV-2 patients [37]. When compared to a control group, these exams showed that plaque scores greater than or equal to 1, gingivitis, mean clinical attachment level greater than or equal to 2, and severe periodontitis were significantly associated with SARS-CoV-2. Probing depths greater than or equal to 4 or 5 mm and clinical attachment levels greater than or equal to 3, 4, or 6 mm were significantly higher in the case group. Both bleeding and plaque accumulation were greater in the case group. These differences in exams between cases and controls highlighted the relationship between periodontitis and more severe SARS-CoV-2 infection. While direct causality cannot be determined by either of these studies, they highlight that periodontal disease is linked to systemic health and may be related to SARS-CoV-2 infection, severity, and complications in direct or indirect mechanisms. Determining periodontal disease extent and severity can help in identifying groups at higher risk of SARS-CoV-2 infection and severity.

Although the data supporting an association between periodontitis and COVID-19 is not definitive, there is biological plausibility for mechanisms that relate these diseases, either from microbial or inflammatory origins. Oral hygiene and periodontal health may play an imperative role in helping prevent SARS-CoV-2 infection, as well as management of severe infections. In addition, oral hygiene measures could potentially prevent the spread of SARS-CoV-2. As discussed previously, the oral cavity can act as a reservoir and point of entry for SARS-CoV-2. SARS-CoV-2 was detected in dental plaque of symptomatic COVID-19 patients [38] and in gingival crevicular fluid and saliva of both asymptomatic and mildly symptomatic COVID patients [39]. These findings should be considered when defining preventive measures to stop the transmission of the SARS-CoV-2 in dental practice.

Oral rinses may provide anti-inflammatory and antiseptic benefits in the oral cavity. A Cochrane review found that rinsing with CHX in addition to oral hygiene for 4-6 weeks to months reduces plaque buildup [40]. CHX is a broad-spectrum antimicrobial agent particularly effective against anaerobes and used as an antiseptic pre- and postprocedural mouth rinse in patients with plaque-induced periodontal diseases. At concentrations of 0.02-0.06%, CHX alters bacterial cell membrane permeability and thus causes cell lysis. It is often used short-term to reduce plaque buildup and gingivitis symptoms and decrease aerosolization of bacteria [41]. A systematic review done by Mohd-Said et al. found that pre-procedural rinses with CHX significantly reduced bacterial contamination post-procedures [42]. Another systematic review that looked into the virucidal efficacy of CHX demonstrated a high virucidal efficacy against Herpes Simplex Type-1 (HSV-1) and Influenza A (InfluA) but lower efficacy against human coronaviruses (HCoVs) including SARS-CoV-2 [43]. Few RCT demonstrated that CHX may temporarily reduce viral load of SARS-CoV-2 in patients with COVID-19. Essential oil-based rinses also have benefits against plaque accumulation and anti-inflammatory effects [44]. Another widely used oral rinse is hydrogen peroxide, an oxidative agent that can reduce viral load [45]. While there is little published evidence on the effects of oral rinses against SARS-CoV-2, multiple studies are ongoing investigating the effects oral rinses may have on patient outcomes and the protection of healthcare workers treating SARS-CoV-2 patients [46]. Due to the stated benefits of oral rinses against other bacteria and viruses, the center for Disease Control and Prevention (CDC) and the American Dental Association (ADA) early on during COVID-19 pandemic recommended pre-procedure mouth rinses of 1.5% hydrogen peroxide of 0.2% povidone in an attempt to reduce viral load and protect healthcare workers [47, 48]. There is a potential benefit of the use of mouth rinses to reduce SARS-CoV-2 viral load. However, the evidence is still limited and inconclusive [49].

## Conclusion

Periodontitis is a chronic inflammatory disease with direct links to multiple systemic diseases, including respiratory infections such as pneumonia. Oral pathogens including periodontal pathogenic bacteria can enter the lung through aspiration in high risk patients resulting in pneumonia. With the emergence of SARS-CoV-2, there is speculation that periodontitis also has direct links to COVID-19 as well. A large body of evidence demonstrated that the oral cavity is a source of pathogens that can disseminate via respiratory, hematologic, and digestive routes. Mounting evidence over the past year found that multiple oral sites, including periodontal pockets, can harbor and replicate SARS-CoV-2, which warrants more research to clarify the links between periodontitis and COVID-19. In addition, oral care measures should be implemented to reduce microbial loads and potentially the transmission of SARS-CoV-2, and the incidence of respiratory infections more broadly.

## Author Contributions

All authors listed have made substantial, direct, and intellectual contribution to the work, and approved it for publication.

## Conflict of Interest

The authors declare that the research was conducted in the absence of any commercial or financial relationships that could be construed as a potential conflict of interest.

## Publisher's Note

All claims expressed in this article are solely those of the authors and do not necessarily represent those of their affiliated organizations, or those of the publisher, the editors and the reviewers. Any product that may be evaluated in this article, or claim that may be made by its manufacturer, is not guaranteed or endorsed by the publisher.

## Abbreviations

COVID-19: Coronavirus Disease 2019
SARS-CoV-2: Severe Acute Respiratory Syndrome Coronavirus 2
TRCs: Taste Receptor Cells
ARDS: Acute Respiratory Distress Syndrome
CRP: C-Reactive Protein.
